# Treatment of Lupus

**Published:** 1860-11

**Authors:** 


### TREATMENT OF LUPUS.

Peter F., aged 17 years, was admitted into the Royal Free Hospital in the first instance, on the 14th of May, 1856, under the care of Mr. Weeden Cooke. His occupation was that of an oyster dredger, at Maldon, in Essex; and he had suffered for seven years from lupoid ulcers of the face, nose, and lips. He had been a patient in the Colchester Hospital, and also in St. George's Hospital. At the time of his admission, the whole face was covered with either the cicatrices of old ulcers, or ulcers encrusted and dipping into the muscles beneath the skin. The columna nasi was destroyed, and the disease was encroaching upon the alæ. The upper lip was thickened and ulcerated. The scaly ulceration extended upwards to the inner canthus of both eyes. Added to these miseries, he was extremely deaf, and altogether presented a most pitiable and unsightly appearance. Mr. Cooke ordered an ounce and half of lemon juice to be taken three times a day, meat and porter, with green vegetables; and the following lotion and ointment: bichloride of mercury, 8 grains; hydrochloric acid, 16 minims; water, 8 ounces; to be applied three times a day as a wash, afterwards covering the parts with zinc ointment. He remained in the hospital under this treatment, gradually improving, until August 17th, when he was discharged, with all the ulcers entirely healed, the deafness diminished, and his general health re-established.

In May, 1857, he again came to town and was admitted, the disease having returned on the cheeks and upper lip. The same treatment was adopted, and this time he was well again in a month.

In July, 1859, he again appeared among Mr. Cooke's outpatients, the upper lip and one cheek only being affected with the scaly ulcers. He was enabled to stay with a sister in town, and therefore not admitted, but placed under the same treatment, namely: lemon juice, fresh green vegetables, bichloride of mercury lotion, and zinc ointment. The cure was very rapid, the ulcers having well cicatrized in less than three weeks. His hearing also at this time was very greatly improved, and his general appearance so altered, that he was scarcely recognized as the same unhappy youth who first came under notice in 1856.

Mr. Cooke states that there was no sponginess of the gums but that the strumous aspect of the lad, and his occupation as an oyster dredger at the sea, led him to prescribe lemon juice as an anti-scorbutic remedy, and, as it turned out, with the happiest results. Still the adjuvant effect of the local applications must be by no means slighted or overlooked in the treatment of similar cases. Perhaps, however, the leaning generally, is to depend too much upon local, and especially caustic applications.—*Lancet.*

